# Gibberellin-Induced Early Flowering of *Cnidium monnieri* Advances the Arrival of Natural Enemies and Increases Their Abundance in Wheat Fields

**DOI:** 10.3390/plants15172567

**Published:** 2026-08-24

**Authors:** Xiaosheng Jiang, Yuanyuan Wang, Guodong Han, Guoxing Gong, Feng Ge, Xingrui Zhang

**Affiliations:** 1 Jinan Engineering Research Center of Ecological Agricultural Products R&D and Industrialization, Institute of Plant Protection, Shandong Academy of Agricultural Sciences, Jinan 250100, China; 2 Shandong Key Laboratory for Green Prevention and Control of Agricultural Pests, Jinan 250100, China; 3 College of Plant Protection, Nanjing Agricultural University, Nanjing 210095, China; 4 College of Agriculture and Forestry Science and Technology, Weifang Vocational College, Weifang 262737, China; 5 College of Plant Protection, Shandong Agricultural University, Tai’an 271018, China

**Keywords:** *Cnidium monnieri*, gibberellin, natural enemies, insectary plant, habitat management

## Abstract

*Cnidium monnieri* (L.) Cusson (Apiaceae) is a well-known insectary plant in farmlands. Its vegetative and flowering stages can promote the migration of natural enemies. However, the arrival of natural enemies in crop fields often lags behind the establishment of pest populations. As gibberellin can accelerate plant growth and flowering, we investigated whether gibberellin treatment could advance the recruitment of natural enemies by altering the phenology of *C. monnieri*. Field experiments were conducted in 2021 and 2022 to evaluate the effects of different gibberellin concentrations on plant phenology, growth traits, and natural-enemy abundance. For field validation, *C. monnieri* strips established in wheat fields were treated with water or 50 mg/L gibberellin. The 50 mg/L treatment advanced the onset of flowering by 21 days and the first detection of natural enemies on *C. monnieri* by 14 days in 2021 and 20 days in 2022. It also significantly increased natural-enemy abundance on *C. monnieri* in 2022. In wheat fields, the same treatment resulted in earlier detection and significantly greater abundance of natural enemies in 2022. The earlier detection of natural enemies was temporally consistent with the advancement of flowering. These findings indicate that manipulating the flowering phenology of insectary plants may improve the timing of natural-enemy establishment and strengthen conservation biological control.

## 1. Introduction

Agricultural intensification has led to changes in farmland landscape patterns and shifts in land cover types. The extensive use of chemical pesticides poses a threat to biodiversity and farmland ecosystems [1,2]. However, prolonged application leads to the development of pesticide resistance and pest resurgence [3,4]. Moreover, the lethal effects of pesticides on natural enemies cannot be overlooked [5]. Additionally, the food safety risks associated with excessive pesticide use remain a major concern. In China, wheat aphid control primarily relies on chemical pesticides. Similar adverse effects of chemical pesticide use, including harm to natural enemies, have also been reported in wheat aphid management in China [6]. Therefore, more environmentally friendly and biologically compatible pest control methods are needed.

Conservation biological control is grounded in ecological principles and aims to protect natural enemies, enhance their persistence and effectiveness, and strengthen the ecosystem services they provide in agroecosystems [7,8]. Habitat management, as the cornerstone of conservation biological control, relies on ecological principles to provide sheltered environments for natural enemies in farmland landscapes. Such management enhances the intensity of biological control and can be achieved through practices such as establishing flower strips [9,10]. More broadly, ecologically based pest management can enhance natural pest regulation by increasing crop and non-crop plant diversity at field and landscape scales [11,12]. Farmland plants provide additional ecological benefits such as boosting biodiversity, supporting natural enemies in controlling pests, improving pollination, reducing pest outbreaks, lowering pesticide use, and increasing crop yield and quality [13,14,15].

The niche and microenvironment within the plant have a significant impact on the habitat and reproduction of insects. Plant physiological and defensive traits can influence the numerical and functional responses of natural enemies; therefore, manipulating these traits may provide a potential means of enhancing biological pest control [16,17]. During the vegetative stage, plant growth regulators influence plant physiology by controlling traits such as plant height, number of stem branches, flower and stem diameter, leaf size and thickness, and stomatal opening and closing [18]. Gibberellins are naturally occurring plant hormones involved in the regulation of seed germination, stem elongation, and flowering. Exogenous gibberellic acid application can promote reproductive development in some plant species, including chrysanthemum and *Paphiopedilum callosum* (Rchb.f.) Stein [19,20,21]. Increasing the availability of suitable habitats and floral resources can enhance the abundance of natural enemies. Therefore, manipulating the growth and flowering traits of functional plants using plant growth regulators may create more favorable habitats for natural enemies while reducing the land area required for their establishment, thereby improving the efficiency and practicality of functional plants in agricultural fields [10,22].

In June 2012, naturally occurring *Cnidium monnieri* (L.) Cusson (Apiaceae) plants along a field margin in Yucheng, Shandong Province, China, were found to harbor abundant predatory natural enemies, particularly *Propylaea japonica* Thunberg and *Harmonia axyridis* Pallas, indicating the potential of this species as an insectary plant for conservation biological control [23,24]. *Cnidium monnieri* produces umbels of successive orders. The primary umbels flower first, followed sequentially by secondary and higher-order umbels, resulting in a prolonged flowering period. Because natural enemies generally enter farmland after pests have become established, the pest-control effects of natural enemies typically occur only after pest populations have begun to increase. To address this issue and enhance the ecological value of *C. monnieri*, we applied gibberellin to alter its flowering period and advance the arrival of natural enemy populations. The objectives of this study were as follows: (i) to determine the effect of gibberellin treatment on the growth, flowering and natural enemy-attracting ability of *C. monnieri*; and (ii) to determine the effects of gibberellin treatment on natural enemy populations when *C. monnieri* is planted as strips in wheat fields.

## 2. Materials and Methods

### 2.1. Overview of the Experimental Design

**Figure 1:**
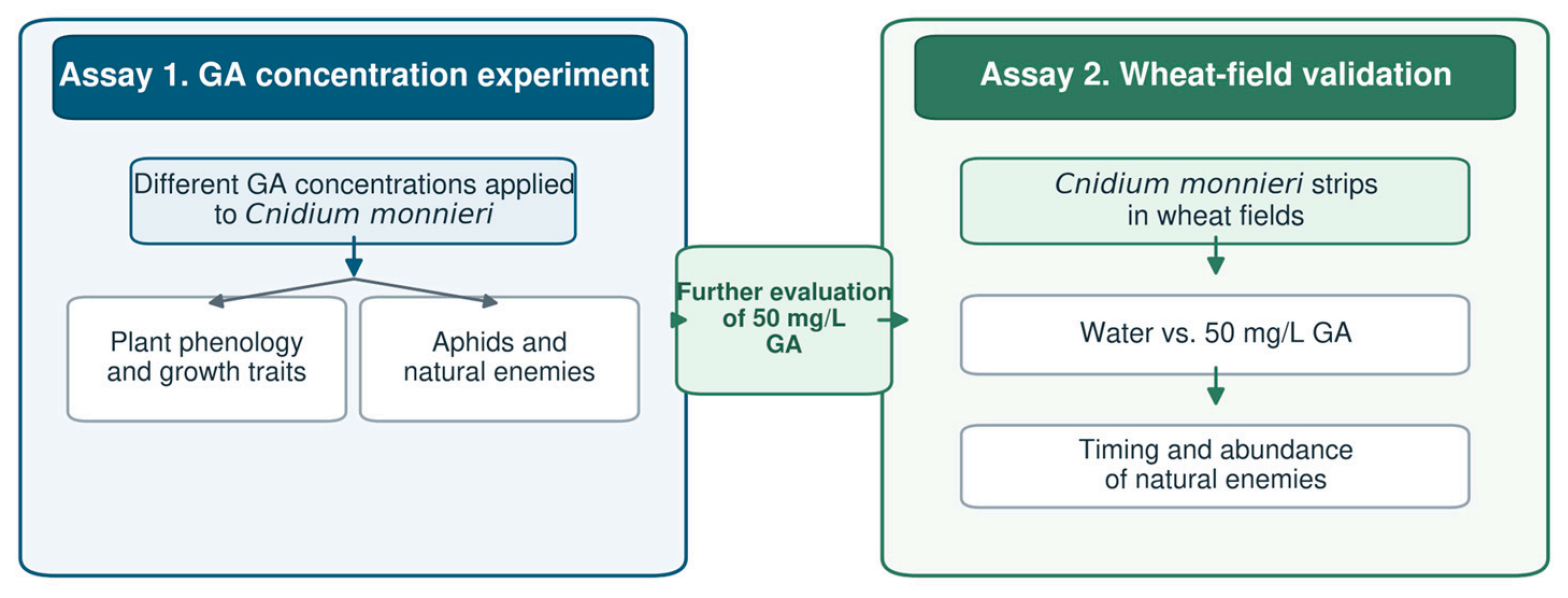
Schematic overview of the two field assays. Assay 1 evaluated the effects of different gibberellin concentrations on the phenology and growth traits of *C. monnieri* and on its associated arthropods. Assay 2 further evaluated the effects of the 50 mg/L gibberellin treatment on the timing and abundance of natural enemies in wheat fields containing *C. monnieri* strips. GA, gibberellin.

The study consisted of two sequential field assays (Figure 1). In the first assay, different gibberellin concentrations were applied to *C. monnieri* to evaluate their effects on plant phenology, growth traits, and the timing and abundance of associated natural enemies. Based on the results of this assay, the 50 mg/L gibberellin treatment was selected for the second assay, in which its effect on natural-enemy establishment was validated using *C. monnieri* strips established in wheat fields. The detailed experimental procedures are described in the following sections.

### 2.2. Experimental Site

The experimental site was located at the Shandong Academy of Agricultural Sciences Experimental Base in Jiyang District, Jinan City, Shandong Province, China (36°58′51″ N, 116°58′23″ E). The experiment was conducted over two consecutive years, 2021 and 2022, at a site managed under a traditional winter wheat–summer maize rotation.

### 2.3. Plant Materials

*Cnidium monnieri* seeds were collected from *C. monnieri*–wheat intercropping fields at the experimental base described above. The seeds were air-dried, threshed, and stored at room temperature until use. The 1000-seed weight was 0.39 g. Gibberellin (purity ≥ 90%; Beijing Solarbio Science & Technology Co., Ltd., Beijing, China) was used in the experiments.

### 2.4. Effect of Gibberellin on Cnidium monnieri Growth

To evaluate the effects of different gibberellin concentrations on the arrival of natural enemies on *C. monnieri*, we established autumn-sown experimental plots in Jinan, Shandong Province, China. These plots were used to investigate changes in the growth stages of *C. monnieri* and the population dynamics of natural enemies and aphids on the plants. Each plot measured 3 m × 3 m and was spaced 3 m apart. The 2021 experiment was conducted as an initial concentration-screening assay using a water control and four gibberellin concentrations (50, 150, 300, and 600 mg/L). Based on the phenological and arthropod responses observed in 2021, the two lower concentrations (50 and 150 mg/L) were retained in 2022 to evaluate the repeatability of their effects. Both concentrations advanced the first detection of natural enemies in 2021, whereas increasing the concentration to 300 or 600 mg/L did not provide a consistent additional benefit across the measured plant and arthropod variables. Therefore, the two higher concentrations were not repeated in 2022.

The seedling stage was defined as the growth stage at which plants had developed 3–5 true leaves, whereas the branching stage was defined by the initiation of lateral branches and the development of 5–7 true leaves; the stem elongation stage was defined by continuous height increase with stem elongation; the budding stage occurred when flower buds emerged at the stem apex; and the flowering initiation stage began with the onset of white flowers. A single plant was considered to have entered the initial flowering stage when the outermost whorl of florets opened. The population was considered to have reached initial flowering when the first *C. monnieri* plant in the group entered this stage; full bloom was defined as when all florets on a single umbel had opened and the petals were fully expanded, and the late flowering stage began when petal wilting became visible.

### 2.5. Effects of Gibberellin Treatment on Natural Enemies

To verify the effects of gibberellin treatment on natural enemy populations, we conducted follow-up experiments in 2022 based on the results obtained in 2021. Gibberellin treatments of 50 and 150 mg/L were retained, and water spray was used as the control. The experiment was arranged in a randomized complete block design with three replicate plots per treatment. Spraying was continued until the leaves were fully wetted. *Cnidium monnieri* was surveyed every 7–9 days from April to August in two consecutive years (2021–2022). Eleven surveys were conducted in 2021 and nine in 2022. Because surveys were conducted at intervals of 7–9 days, the arrival time of natural enemies was operationally defined as the date on which natural enemies were first detected during the surveys. The five-point survey method was used, with each survey point consisting of a 0.5 m × 0.5 m sampling frame. Plants within the frame were evaluated, and the main natural enemies were recorded in 0.25 m^2^.

In 2021 and 2022, *C. monnieri* was planted in wheat fields, with a 1 m wide strip of *C. monnieri* established every 30 m. Spraying was applied as described above. Gibberellin was applied only to the *C. monnieri* strips and was not sprayed directly onto the wheat plants. No insecticides were applied in the experimental plots after the establishment of *C. monnieri*. Natural enemies and celery aphids, *Semiaphis heraclei* Takahashi, were counted every 7–9 days from April to August using the same five-point survey method. At each survey point, a 0.5 m × 0.5 m sampling frame was used; plants within the frame were examined, and the main natural enemies were counted within the 0.25 m^2^ area.

### 2.6. Data Analysis

Plant height at each growth stage and umbel diameter at each flowering stage were analyzed separately using one-way analysis of variance (ANOVA), followed by Tukey’s HSD test for multiple comparisons among treatments. Natural-enemy and aphid abundance data were analyzed using generalized linear mixed models. For the 2021 data, the fixed effects were treatment (water and 50, 150, 300, and 600 mg/L gibberellin), sampling date (11 dates), and their interaction. For the 2022 data, the fixed effects were treatment (water, 50 mg/L gibberellin, and 150 mg/L gibberellin), sampling date (9 dates), and their interaction. Block was included as a random effect. Because aphids were rarely observed in 2022 and counts were zero at almost all sampling points, the 2022 aphid data were not subjected to inferential statistical analysis because of insufficient variation. The ‘emmeans’ package was used for pairwise post hoc comparisons among treatments within each year. The significance of fixed effects in the generalized linear mixed model was assessed using Type II Wald chi-square tests with the Anova function in the car package. All statistical analyses were performed using R version 4.2.0 (R Core Team, Vienna, Austria, 2022).

## 3. Results

### 3.1. Effects of Gibberellin on the Phenology and Growth Traits of Cnidium monnieri

**Figure 2:**
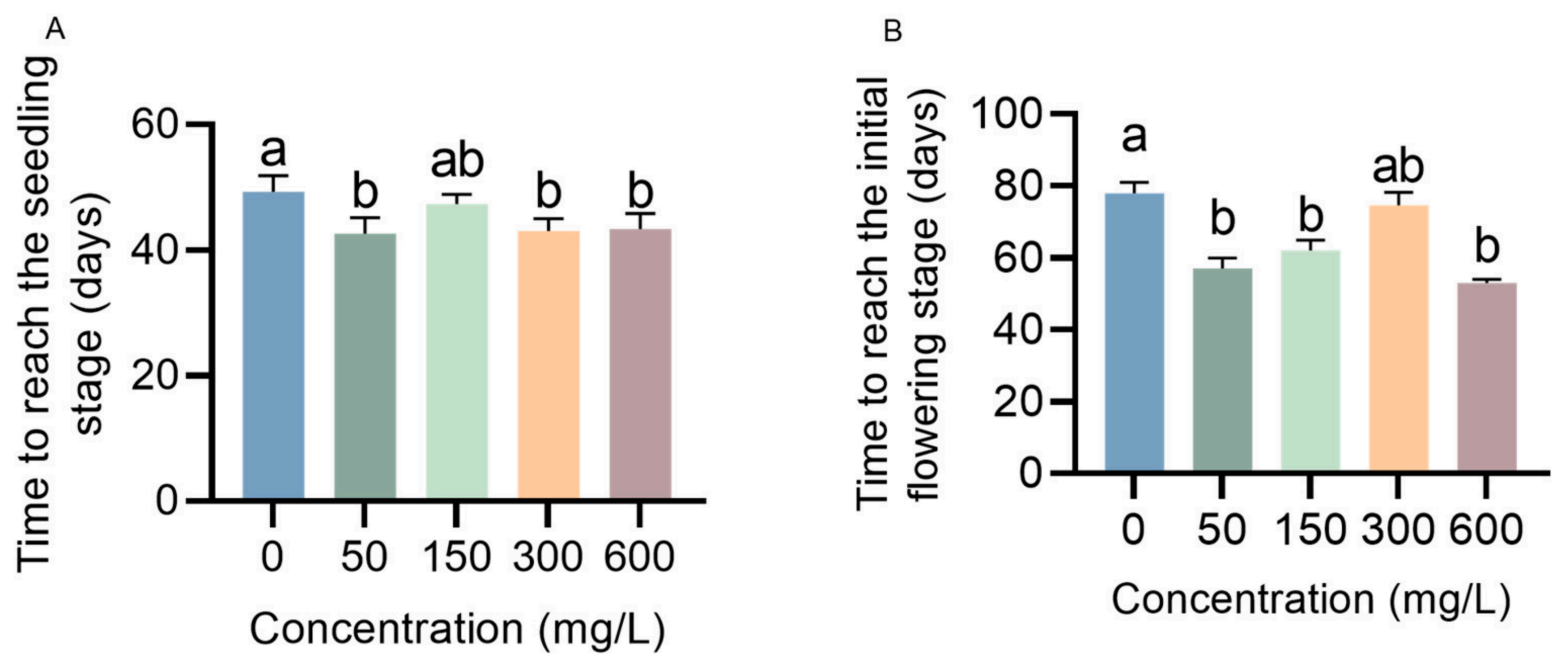
Effect of gibberellin treatment on the growth period of *C. monnieri*. (**A**): Time required for *C. monnieri* treated with different gibberellin concentrations to reach the seedling stage; (**B**): time required for *C. monnieri* treated with different gibberellin concentrations to reach the initial flowering stage. Different lowercase letters indicate significant differences among treatments at *p* < 0.05. Bars represent means ± SE.

Measurements of *C. monnieri* phenology showed that untreated control plants required 49 days from sowing to reach the seedling stage (Figure 2A) and 78 days to reach the initial flowering stage (Figure 2B). Gibberellin application shortened the growth period. Treatment of *C. monnieri* with 50 mg/L gibberellin led to a 6-day advance in the seedling stage and a 21-day advance in the flowering stage compared to the control. The time required to reach the seedling stage was significantly shorter under the 50 mg/L gibberellin treatment than under the other treatments (Figure 2A). Similarly, the 50 mg/L gibberellin treatment significantly shortened the time to initial flowering (Figure 2B).

**Figure 3:**
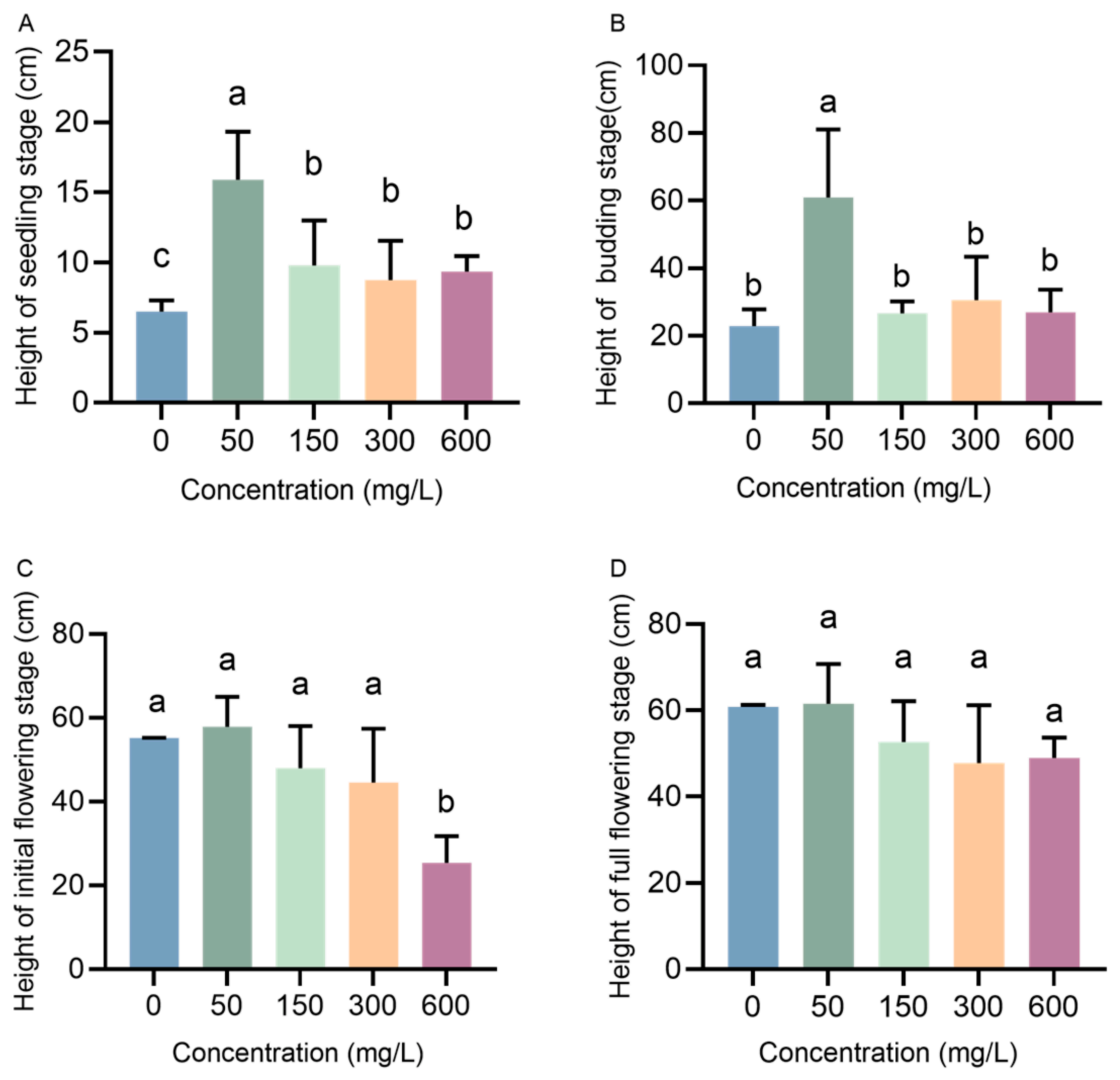
Effect of gibberellin treatment on the height of *C. monnieri* at different growth stages: (**A**): seedling stage; (**B**): budding stage; (**C**): initial flowering stage; and (**D**): full flowering stage. Different lowercase letters indicate significant differences among treatments at *p* < 0.05. Bars represent means ± SE.

Plant height of *C. monnieri* was measured at four growth stages. At the seedling stage, the mean plant height in the water control was 6.50 cm, and the 50 mg/L gibberellin treatment significantly increased plant height compared with the water control (Figure 3A). At the bud stage, the mean plant height in the water control was 22.83 cm, and plant height was also significantly increased by the 50 mg/L gibberellin treatment (Figure 3B). At the initial flowering stage, the mean plant height in the water control was 55.13 cm, with no significant difference between the 50 mg/L gibberellin treatment and the water control (Figure 3C). Similarly, at the full flowering stage, the mean plant height in the water control was 60.78 cm, and no significant difference was detected between the 50 mg/L gibberellin treatment and the water control (Figure 3D).

**Figure 4:**
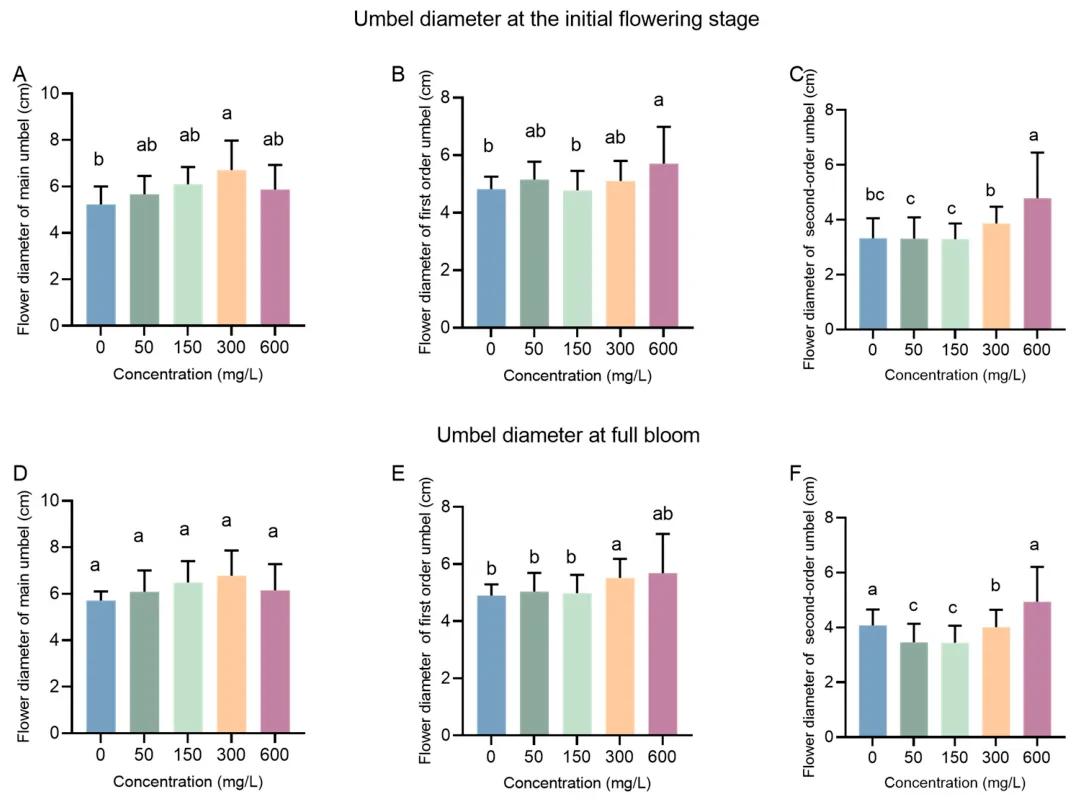
Effects of different gibberellin concentrations on the diameter of *C. monnieri* umbels at different flowering stages. (**A**–**C**) Umbel diameter at the initial flowering stage: (**A**) main umbel, (**B**) first-order umbel, and (**C**) second-order umbel. (**D**–**F**) Umbel diameter at full bloom: (**D**) main umbel, (**E**) first-order umbel, and (**F**) second-order umbel. Bars represent means, and error bars indicate SE. Different lowercase letters indicate significant differences among treatments within each panel, as determined by one-way ANOVA followed by Tukey’s HSD test (*p* < 0.05).

At the initial flowering stage, the mean diameters of the main and first-order umbels of *C. monnieri* were 5.22 and 4.82 cm, respectively (Figure 4A,B). Gibberellin treatments generally increased umbel diameter; however, the increases under the 50 mg/L gibberellin treatment were not significant (Figure 4A–C). At the full flowering stage, the mean diameters of the main and first-order umbels were 5.71 and 4.90 cm, respectively (Figure 4D,E).

### 3.2. Effects of Gibberellin Treatment on Natural Enemies in Cnidium monnieri

Based on two years of field investigations, the main natural enemies observed on *C. monnieri* were *H. axyridis*, *P. japonica* and *Orius* spp. ISyrphidae and Chrysopidae were also frequently observed; however, they were excluded from the analysis because only adults were recorded on *C. monnieri*, whereas the larvae represent the principal predatory stages responsible for pest suppression. Natural enemies generally began to migrate in spring, corresponding to the bud stage of *C. monnieri*. Different natural enemies emerged at different times. Lady beetles appeared first and remained on *C. monnieri* throughout the survey period. During the flowering period, *Orius* spp. began to emerge and colonize *C. monnieri*. The timing of the first detection of natural enemies was recorded. 

Generalized linear mixed models showed that predator abundance in 2021 was significantly affected by sampling date, but not by gibberellin treatment or the treatment × date interaction. In 2022, predator abundance was significantly affected by gibberellin treatment, sampling date, and their interaction. Aphid abundance in 2021 was significantly affected by sampling date and the treatment × date interaction, but not by gibberellin treatment (Table 1). Aphids were rarely observed in 2022, with zero counts at almost all sampling points; therefore, no inferential statistical analysis was conducted for aphid abundance in that year.

**Figure 5:**
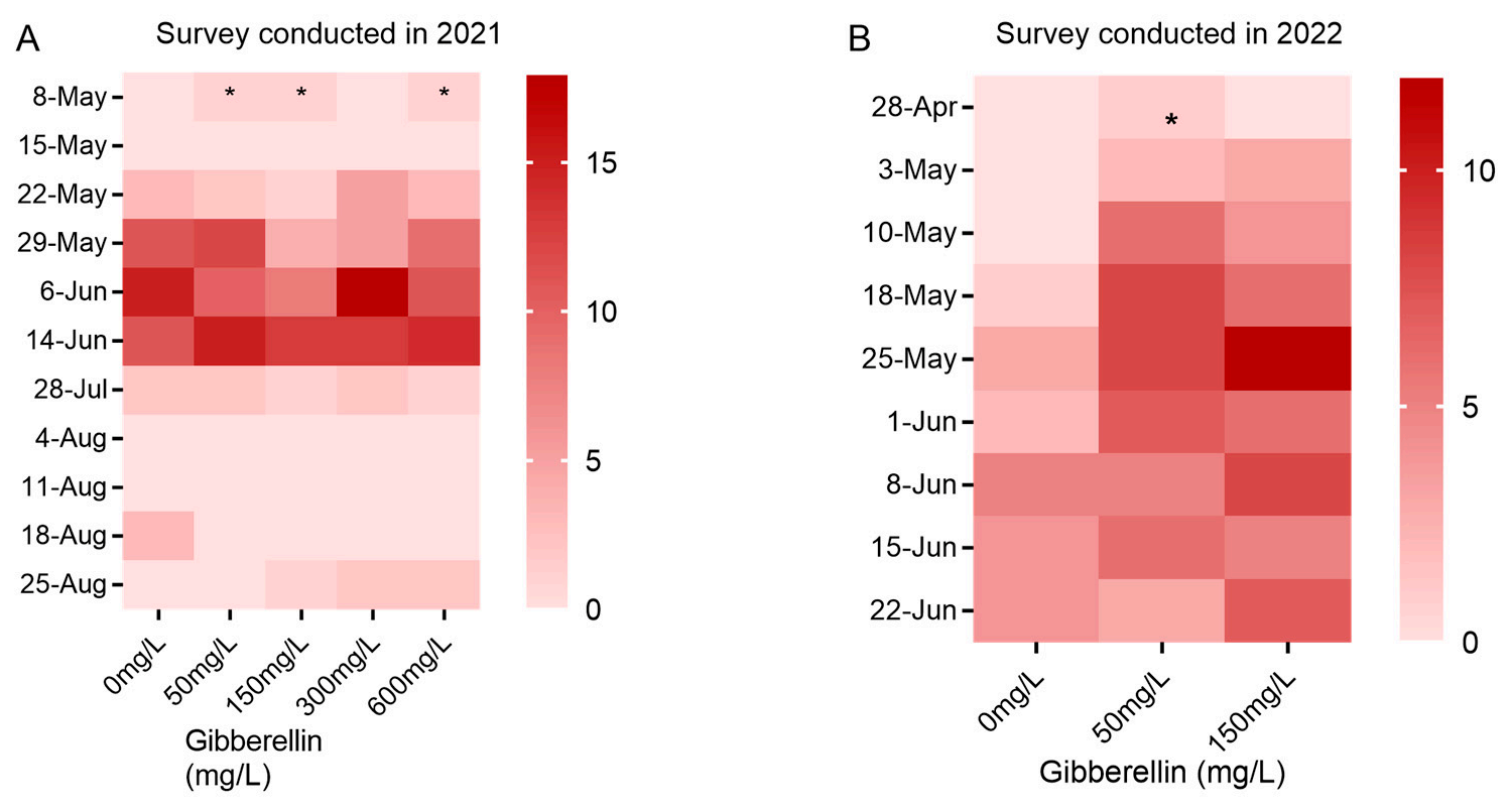
Temporal dynamics of natural enemy abundance on *Cnidium monnieri* under different gibberellin treatments in 2021 and 2022. (**A**) Survey conducted in 2021; (**B**) survey conducted in 2022. Treatment codes are as follows: The color scale indicates natural enemy abundance (individuals per 0.25 m^2^), with darker colors representing higher abundance. Asterisks indicate the first detection of natural enemies under treatments that advanced their appearance relative to the water control.

The timing of the first detection of predators differed among gibberellin treatments. In 2021, predators were first detected on 22 May in the water-control and 300 mg/L treatment plots, whereas they were first detected on 8 May in the 50, 150, and 600 mg/L treatment plots, representing an advance of 14 days. In 2022, predators were first detected on 18 May in the water-control plots, 28 April in the 50 mg/L treatment plots, and 3 May in the 150 mg/L treatment plots, representing advances of 20 and 15 days, respectively (Figure 5).

In 2021, the 50 mg/L gibberellin treatment numerically increased predator abundance, although the treatment effect was not statistically significant (Table 1 and Figure 5A). In 2022, predator abundance differed significantly among gibberellin treatments, and the 50 mg/L treatment resulted in greater predator abundance than the water control (Table 1 and Figure 5B).

In 2021, 50 mg/L gibberellin increased natural enemy abundance, but the increase was not statistically significant. In 2022, treatment with 50 mg/L gibberellin significantly increased the natural enemy population size (Table 1). Aphids were rarely observed in 2022, with zero counts recorded at almost all sampling points; therefore, no inferential statistical analysis was conducted for aphid abundance in that year. Treatment with 50 mg/L gibberellin advanced the observed arrival time and increased the population size of natural enemies. Since the hormone treatment changed plant traits, it was speculated that gibberellin treatment changed the physiological characteristics of *C. monnieri* and thus influenced the dynamics of natural enemies.

### 3.3. Effects of Gibberellin Treatment on Natural Enemies in Wheat Fields

**Figure 6:**
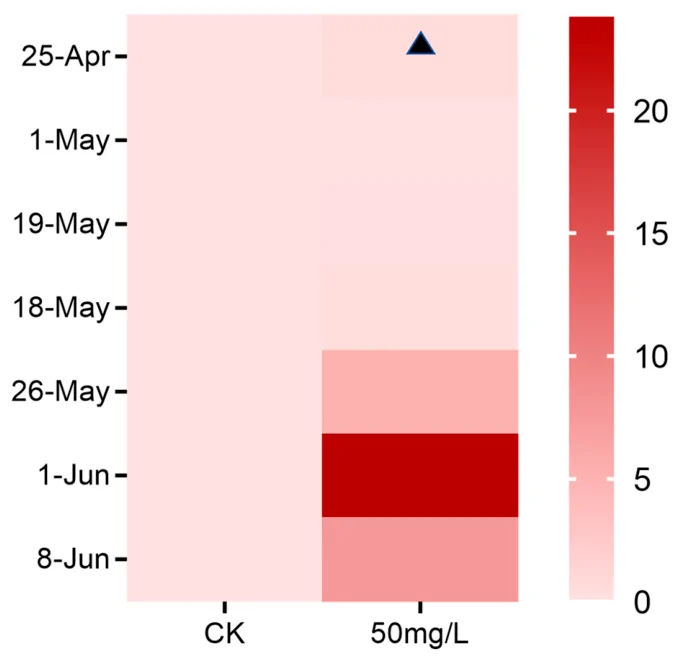
Temporal dynamics of natural enemy abundance in wheat fields adjacent to *Cnidium monnieri* strips treated with water or 50 mg/L gibberellin in 2022: The color scale indicates natural enemy abundance (individuals per 0.25 m^2^), with darker colors representing higher abundance. The triangle indicates the first detection of natural enemies in the 50 mg/L gibberellin treatment, which occurred earlier than that in the water control.

In 2021, gibberellin treatment increased natural enemy abundance, although the increase was not statistically significant. In 2022, the abundance of natural enemies in wheat fields was significantly higher under the 50 mg/L gibberellin treatment than under the water control (*p* = 0.004), and natural enemies were detected earlier in the treated plots (Figure 6).

## 4. Discussion

Habitat loss, invasive pest species, and destructive exploitation of resources have led to massive losses of insect diversity over the past 100 years [25]. Against this background, ecologically based pest management has received increasing attention, with functional plants increasingly being incorporated into agricultural landscapes to regulate plant–herbivore–natural enemy interactions, enhance biological pest control, and support other ecosystem services [26,27]. In farmland ecosystems, selecting suitable functional plants from among flowering species to provide habitat, reproductive sites, and food resources for predators has become an important research focus [28]. For example, planting rapeseed around wheat fields can control wheat aphids in adjacent fields by attracting syrphid flies [29]. Intercropping alfalfa and wheat can control wheat aphids by attracting natural enemies, such as lady beetles and parasitic wasps [30]. Trap crops can effectively suppress lepidopteran stemborers in cereal systems. For example, Napier grass and Sudan grass planted around maize fields attract cereal stemborers away from maize in eastern Africa, while vetiver grass planted along paddy-field margins acts as a dead-end trap crop for *Chilo suppressalis* Walker [31,32]. 

This study showed that exogenous gibberellin altered the developmental timing of *C. monnieri* and was associated with the earlier detection of natural enemies. Among the concentrations tested, the 50 mg/L treatment produced the most consistent response. It advanced the initial flowering of *C. monnieri* by 21 days and advanced the first detection of natural enemies by 14 days in 2021 and 20 days in 2022. In the wheat-field validation experiment, the same treatment also increased natural-enemy abundance relative to the water control. These findings indicate that low-concentration gibberellin treatment may help reduce the temporal mismatch between floral-resource availability and the early-season occurrence of natural enemies in wheat fields. The microenvironment has a complex and significant impact on the composition and movement of insects [33,34,35]. The leaves and flowers of plants form a microenvironment that is influenced by factors such as plant height, flower diameter, and leaf characteristics. A complex and diverse environment can provide more ecological niches that are conducive to the shelter and reproduction of predators.

The effects of gibberellin were not consistently enhanced by increasing its concentration. The 50 mg/L treatment shortened the time required for *C. monnieri* to reach the seedling and initial flowering stages and increased plant height during the early growth stages. However, it did not significantly affect plant height at the initial or full flowering stage or the diameters of the different umbel orders. These results suggest that the principal effect of the 50 mg/L treatment was an acceleration of early plant development rather than a persistent increase in final plant size or umbel diameter. The absence of consistent additional benefits at higher concentrations also supports the selection of 50 mg/L for further field evaluation. Plant architecture is an important determinant of predator attraction, and different predators occupy different niches. For example, apple-tree canopy structure modifies insect microhabitats and thereby affects insect distribution [36]. Leaf surface temperature and stomatal opening and closing also have an important influence on the distribution of aphids [35]. For example, pine processionary moths create a habitable environment by generating heat [37]. Therefore, the shelter environment provided by the plant itself and the appropriate microenvironment have a significant impact on the migration of natural enemies.

Exogenous plant growth regulators can affect endogenous hormones and volatile substances released by plants and may even affect the Bt protein content in Bt varieties [16,38]. Foliar application of methyl jasmonate to cotton reduced the abundance of sucking insect pests and marginally increased the abundance of some associated predators [39]. In rice, exogenous application of jasmonic acid induces volatile emissions and enhances the parasitism of brown planthopper eggs by the egg parasitoid *Anagrus incarnatus* Haliday [40]. Similarly, exogenous application of methyl jasmonate and salicylic acid to citrus alters foliar volatile emissions and influences the aggregation behavior of the Asian citrus psyllid, *Diaphorina citri* Kuwayama. Methyl jasmonate treatment also increases the emission of volatile compounds that are potentially attractive to the psyllid’s natural enemies [41]. Although earlier detection of natural enemies was observed following gibberellin treatment in both years, the response of their abundance differed between years. In 2021, the overall treatment effect on predator abundance was not significant, whereas significant effects of treatment, sampling date, and their interaction were detected in 2022. Therefore, the effect of gibberellin was more consistent for the timing of natural-enemy detection than for their overall abundance during the two-year concentration experiment. Interannual differences in weather conditions, prey availability, and plant development may have contributed to this variation. In particular, aphids were rarely observed in 2022, which may have altered the relative importance of floral resources for natural enemies.

In the present study, *H. axyridis* occurred mainly beneath the umbels of *Cnidium monnieri*, whereas *P. japonica* occurred beneath both the umbels and leaves. Overlapping niches may lead to competition among predators through intraguild predation and competition for shared prey [42,43]. These interactions may alter predator community composition and influence their dispersal from *C. monnieri*. On *C. monnieri* plants colonized by aphids, *H. axyridis* and *P. japonica* were the dominant predators, whereas *Orius* spp. were rarely observed. Because lady beetles consume large numbers of aphids, depletion of aphid prey on *C. monnieri* may encourage their dispersal into adjacent crop fields in search of additional prey [23,44]. By consuming scattered aphid colonies, lady beetles may suppress early aphid population growth. The aphid population on *C. monnieri* may therefore be insufficient to sustain lady beetle populations throughout the summer, potentially encouraging their movement into adjacent fields [23,44]. Because gibberellin application was confined to *C. monnieri* under insecticide-free conditions, this approach may provide a practical means of enhancing natural-enemy conservation without directly applying the growth regulator to wheat.

In this study, by applying gibberellin to *C. monnieri*, we found that this treatment could regulate the timing at which *C. monnieri* attracted natural enemies, promote the early movement of natural enemies into farmland for pest control, and enhance the natural-enemy conservation capacity of *C. monnieri*, thereby facilitating their dispersal from *C. monnieri* into adjacent farmland. These findings suggest that manipulating the flowering phenology of *C. monnieri* with gibberellin may be a promising strategy for strengthening conservation biological control by enhancing the ecological pest-control function of functional plants, supporting the regulation of natural-enemy dynamics, and potentially reducing reliance on pesticides.

## Figures and Tables

**Table 1:** Effects of gibberellin treatment, sampling date, and their interaction on predator and aphid abundance.

Response Variable	Year	Fixed Effect	df	χ^2^	*p*
Predator abundance	2021	Treatment	4	6.844	0.144
		Date	10	757.129	<0.001
		Treatment × Date	40	47.365	0.197
	2022	Treatment	2	39.980	<0.001
		Date	8	231.596	<0.001
		Treatment × Date	16	58.564	<0.001
Aphid abundance	2021	Treatment	4	4.161	0.385
		Date	10	85.845	<0.001
		Treatment × Date	40	69.896	<0.001

Note: The significance of fixed effects was assessed using Type II Wald chi-square tests. Block was included as a random effect. Aphid abundance is presented only for 2021 because aphids were rarely observed in 2022 and counts were zero at almost all sampling points, resulting in insufficient variation for inferential statistical analysis.

## Data Availability

The raw data supporting the conclusions of this article will be made available by the authors on request.
